# Passage of an Anatomy Act in Pennsylvania

**Published:** 1867-05

**Authors:** 


### PASSAGE OF AN ANATOMY ACT IN PENNSYLVANIA.

Within the last week the Legislature of this State has passed the following important act, which, it is understood, will receive the approval of the Governor:

*An Act for the Promotion of Medical Science, and to Prevent the Traffic in Human Bodies.*

SECTION 1. Be it enacted by the Senate and House of Representatives of the Commonwealth of Pennsylvania, in General Assembly met, and it is hereby enacted by the authority of the same, that, any public officer having charge or control over the same, within the limits of Philadelphia and Allegheny Counties, shall give permission to any Physician or Surgeon of the same county, upon his request made therefor, to take the bodies of such persons as are required to be buried at the public expense, to be by him used within the State for the advancement of Medical Science, preference being given to Medical Schools, public and private; and said bodies to be distributed to and among the same, equitably, the number assigned to each being proportioned to that of its students; provided, however, that if the deceased person, during his or her last sickness, of his or her own accord, shall request to be buried; or if any person claiming to be, and satisfying the proper authorities that he is of kindred to the deceased, shall ask to have the body for burial, it shall be surrendered for interment; or, if such deceased person was a stranger or traveller, who died suddenly, the body shall be buried, and shall not be handed over as aforesaid.

SECTION 2. Every Physician or Surgeon, before receiving any such dead body, shall give to the proper authorities surrendering the same to him, a sufficient bond that each body shall be used only for the promotion of Medical Science within this State, and whosoever shall use such body or bodies for any other purpose, or shall remove the same beyond the limits of this State; and whosoever shall sell or buy such body or bodies, or in any way traffic in the same, shall be deemed guilty of a misdemeanor, and shall, on conviction, be imprisoned for a term not exceeding five years, at hard labor in a county jail.

Similar legislation has been three times, at least, urged upon our Legislature before, unsuccessfully. Misunderstanding, prejudice, and technical delays, have defeated it. This time, the College of Physicians, of Philadelphia, upon the motion of Dr. W. S. Forbes, gave its official sanction to the effort. A committee, consisting of Drs. Forbes, Agnew, and H. Hartshorne, visited Harrisburg on behalf of the College. Especially through the exertions of Senator Wilmer Worthington, M.D., the act was rescued from burial in Committee, and passed through the Senate. In the House, it was called up by Mr. Freeborn, and passed within a few days. Thus one of the obstacles to medical teaching in Philadelphia is removed, and we are, in this respect, on the same footing as New York. It is a not uninteresting fact, that the passage of a similar bill, the "Warburton Act," by the British Parliament, more than forty years ago, followed the agitation resulting from the discovery of the murders perpetrated by Burke and Hare to obtain subjects for sale, at lower prices than, from want of this bill, they have lately commanded here.

